# Clinical Potential of Artificial Bone Scintigraphy from Early-Phase Bone Scintigraphy Using Unpaired Image-to-Image Translation in Patients with Breast Cancer: A Single-Center Prospective Study

**DOI:** 10.3390/tomography12040050

**Published:** 2026-04-02

**Authors:** Yong-Jin Park, Il-Hyun Kim, Young-Sil An, Joon-Kee Yoon, Su Jin Lee

**Affiliations:** 1 Department of Nuclear Medicine, Ajou University Medical Center, Ajou University School of Medicine, 164 Worldcup-ro, Yeongtong-gu, Suwon 16499, Republic of Korea; yongjin.park@hanmail.net (Y.-J.P.); aysays@aumc.ac.kr (Y.-S.A.); jkyoon3@aumc.ac.kr (J.-K.Y.); 2 Department of Nuclear Medicine, Seoul National University Hospital, 101 Daehak-ro, Jongno-gu, Seoul 03080, Republic of Korea; titan31415@naver.com; 3 Department of Nuclear Medicine, Soonchunhyang University Bucheon Hospital, 170, Jomaru-ro, Bucheon 14584, Republic of Korea

**Keywords:** breast cancer, bone scintigraphy, unpaired image-to-image translation, contrastive unpaired translation

## Abstract

**Objectives**: The objective of this study is to investigate the clinical potential of generating artificial bone scintigraphy (aBS), defined here as a deep learning-generated bone scintigraphy image that simulates delayed-phase bone scintigraphy (dBS) characteristics, from early-phase bone scintigraphy (eBS) obtained with a short waiting time using an unpaired image-to-image translation method in patients with breast cancer (BC). **Methods**: In this single-center prospective study involving 245 patients with BC (195 for training and 50 for testing), eBS and dBS were performed. Using the contrastive unpaired translation (CUT) model, we trained with anterior and posterior images of the eBS and dBS from the training group. We then generated aBS images targeting dBS by inputting eBS from the test group for both anterior and posterior views. We conducted quantitative, qualitative, and visual assessments to evaluate aBS. **Results**: The points of the anterior and posterior images of aBS on the qualitative four-point and five-point rating scales were significantly higher than those of eBS (*p* < 0.0001). Three nuclear medicine physicians performed visual assessments, demonstrating consistent findings on the presence of bone metastases in both aBS and dBS. Their visual evaluations indicated that the bone-to-soft tissue contrast in aBS was superior to that in eBS. The quantitative metrics of aBS were superior to those of eBS. However, aBS was inferior to the targeted dBS in terms of qualitative and visual assessments. **Conclusions**: The aBS generated through CUT was superior to eBS in quantitative, qualitative, and visual assessments and preserved lesion-related information comparable to dBS. Although these findings do not support replacement of dBS for definitive diagnosis, they support the feasibility of aBS as an assistive delayed-phase-like image generation approach from earlier-acquired bone scintigraphy.

## Simple Summary

Bone scintigraphy (BS) is widely used to detect bone metastases in patients with breast cancer; however, conventional delayed-phase bone scintigraphy (dBS) requires a prolonged waiting time after radiotracer injection. This study evaluated whether artificial intelligence (AI) could generate images comparable to dBS using early-phase bone scintigraphy (eBS) acquired shortly after injection. Artificial bone scintigraphy (aBS) images demonstrated improved bone-to-soft tissue contrast and overall image quality compared with eBS while preserving lesion-related information comparable to dBS. Although these findings do not support replacement of dBS for definitive diagnosis, they support the feasibility of aBS as an assistive delayed-phase-like image generation approach from earlier-acquired BS and provide a basis for future studies evaluating its clinical utility.

## 1. Introduction

Bone scintigraphy (BS) is a widely performed examination in nuclear medicine and plays a crucial role in detecting bone metastases (BMs) in patients with breast cancer (BC) [1,2]. BC is the most prevalent cancer among women worldwide [3]. Bone, particularly the axial skeleton, is the most common site for distant metastases in BC [4]. Approximately half of patients with advanced BC develop BMs, and approximately 70% of patients who die from BC experience BMs [4]. Therefore, early and accurate diagnosis of BM in patients with BC is crucial for improving treatment outcomes and prognosis [5]. In a previous meta-analysis on BM in BC, the pooled estimates for the sensitivity and specificity of BS were 0.93 and 0.86, respectively [5]; these results highlight BS as a crucial examination for evaluating BMs in patients with BC. Delayed-phase bone scintigraphy (dBS) is typically performed after a long waiting time of 2–5 h following intravenous injection of a bone-seeking radiotracer [1]. This long waiting time is necessary to allow sufficient clearance of the radiotracer from the blood and soft tissues, thereby optimizing the bone-to-background ratio [1,6,7]. However, the prolonged waiting time required for dBS imaging has been a source of patient discomfort and is considered a bottleneck for patient flow in nuclear medicine departments with a limited number of gamma cameras [8]. Therefore, there is a need for BS that can be obtained with a shorter waiting time while maintaining image quality and avoiding missing lesions due to a reduced bone-to-soft tissue ratio.

Given that the aim of image-to-image (I2I) translation is to transform an input image from a source domain to a target domain while preserving the intrinsic content of the source and incorporating the external style of the target [9], this suggests the potential to generate artificial bone scintigraphy (aBS), defined in this study as a deep learning-generated bone scintigraphy image that simulates the imaging characteristics of dBS while preserving structural information from early-phase bone scintigraphy (eBS). Until now, the shorter waiting time of eBS has been weighed against the trade-offs involving image quality, optimal bone-to-soft tissue ratio, and clinical information provided by dBS. Khan et al. compared eBS obtained 1.5 h after technetium-99m (Tc-99m) methylene diphosphonate (MDP) injection with dBS obtained 3 h post-injection [8]. They reported that eBS exhibited fair bone-to-soft-tissue contrast, while dBS exhibited good bone-to-soft-tissue contrast [8]. Although eBS and dBS have different imaging characteristics owing to different waiting times, we hypothesized that using I2I translation could generate an aBS that combines the short waiting time of eBS with the imaging characteristics of dBS. Among the I2I translation methods, the paired I2I approach uses aligned image pairs from the source and target domains to transform the source image into the desired target image [9]. Park et al. trained the pix2pix model, a paired I2I translation method, using blood pool and delayed images from a three-phase BS for complex regional pain syndrome (CRPS), inflammatory arthritis, cellulitis, osteomyelitis, and recent bone injury [10]. The trained pix2pix model generated delayed images from blood pool images with hyperemia, and the sensitivities of the generated images for CRPS and inflammatory arthritis were 87.5% and 77.8%, respectively [10]. However, training using paired I2I translations is impractical because gathering large amounts of paired training data is costly and challenging [9]. Additionally, because of the different patient positions at the time of imaging for eBS and dBS, which are obtained at different times, unpaired I2I translation methods are more suitable than paired I2I translation methods. Contrastive unpaired translation (CUT) is an unpaired I2I translation method that preserves the structure of an input patch while incorporating the appearance or texture of the target patch [11]. Therefore, we speculated that by training the CUT model with eBS and dBS, we could generate an aBS targeting dBS using eBS as input data.

In this single-center prospective study on patients with BC, we hypothesized that generating aBS from eBS using the CUT model would produce aBS images with the imaging characteristics of dBS. After training the CUT model with a training group consisting of eBS and dBS, we generated an aBS targeting dBS by using eBS from the test group as input. Quantitative, qualitative, and visual assessments were performed to evaluate aBS in the test group. Through these assessments, we verified whether aBS exhibits the imaging characteristics of dBS and analyzed the clinical potential of aBS compared to eBS.

## 2. Materials and Methods

### 2.1. Study Population

We prospectively collected both eBS and dBS data from 360 consecutive patients with BC who provided informed consent between August 2020 and October 2021 at Ajou University Medical Center. Patients (i) with cancers other than BC (*n* = 95), (ii) suspected rheumatic or joint disease (*n* = 15), or suspected trauma (*n* = 5) were excluded. Finally, 245 patients with BC who underwent both eBS and dBS were enrolled. We randomly assigned 245 patients with BC into two groups (training group, 195; test group, 50). A simple random split was used for dataset allocation without prespecified age-based stratification. The Institutional Review Board of Ajou University Medical Center approved the prospective data collection protocol (approval no. AJOUIRB-OBS-2020-281). All procedures were conducted in accordance with the 2013 Declaration of Helsinki and its subsequent amendments, and similar ethical standards.

### 2.2. Acquisition Protocol for Bone Scintigraphy

The patients underwent planar whole-body BS after an intravenous injection of 740 MBq (20 mCi) Tc-99m hydroxymethylene diphosphonate (HMDP). Four different dual-head gamma cameras, including Infinia Hawkeye 4 (GE Healthcare, Milwaukee, WI, USA), Symbia E (Siemens Healthcare, Erlangen, Germany), Discovery NM 830 (GE Healthcare, Milwaukee, WI, USA), and Discovery NM/CT 670 (GE Healthcare, Milwaukee, WI, USA), were used to obtain BS images. The scan speed and pixel size of the gamma cameras were 18 cm/min and 2.21 mm, respectively. All gamma cameras used a low-energy, high-resolution collimator, and the matrix size of all BS was set to 256 × 1024. eBS and dBS images were obtained approximately 80 and 200 min after radiotracer injection, respectively.

### 2.3. Unpaired Image-to-Image Translation Method

In this study, an unpaired I2I translation method was used because of inconsistencies in the imaging positions of patients with BC between eBS and dBS. We utilized the official PyTorch implementation of CUT to generate aBS, which in this study refers to a deep learning-generated BS image intended to simulate dBS characteristics while preserving structural information from eBS. Except for the study-specific settings explicitly described below, we used the default architecture and training configuration provided in the official CUT implementation [11]. This approach enables the learning of mapping functions between two different domains using unpaired image datasets. In the CUT framework, the generator objective included an adversarial least-squares generative adversarial network (GAN) loss and a patch-wise contrastive loss (PatchNCE) [11]. During training, the generator-side optimization dynamics, including the overall generator loss (G), adversarial generator loss (G_GAN), and PatchNCE loss (NCE), were monitored using the built-in logging utilities of the official CUT framework (Figure S1). The CUT maintains the structure of the input patch while considering the appearance or texture of the target patch by maximizing the mutual information between the corresponding input and output patches [11]. Therefore, we aimed to generate an aBS that retains the structure of an eBS while incorporating the appearance or texture of a dBS. The open-source code for the CUT model is available at https://github.com/taesungp/contrastive-unpaired-translation (accessed on 14 March 2022) [11].

The generator was a ResNet-based generator with nine residual blocks (netG = resnet_9blocks; ngf = 64), whereas the discriminator was a 70 × 70 PatchGAN discriminator (netD = basic; n_layers_D = 3; ndf = 64); both networks used instance normalization. For contrastive learning, the feature sampling network was configured as mlp_sample with a 256-dimensional projection head (netF_nc = 256). PatchNCE loss was computed at layers 0, 4, 8, 12, and 16 using 256 sampled patches per layer and a temperature parameter of 0.07.

**Figure 1:**
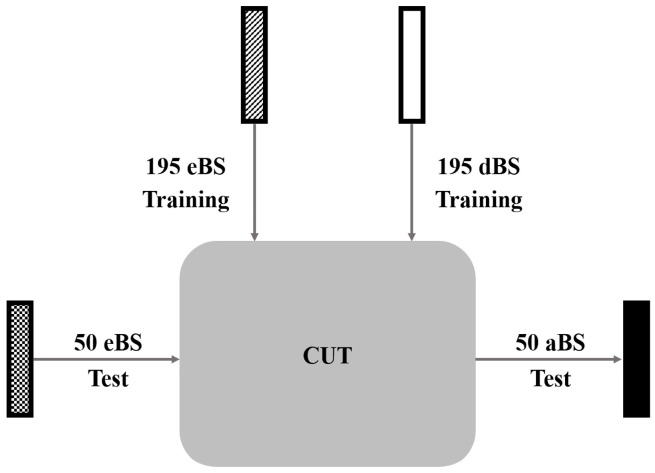
Schematic diagram of aBS generation using an unpaired I2I translation method. In this study, aBS denotes a deep learning-generated BS image simulating dBS characteristics from eBS input. We trained the model using the CUT method, utilizing 195 eBS and 195 dBS as training datasets. Using the trained CUT model, we inputted 50 eBS from the test datasets and obtained 50 aBS as the output. Abbreviations: aBS, artificial bone scintigraphy; I2I, image-to-image; BS, bone scintigraphy; dBS, delayed-phase bone scintigraphy; eBS, early-phase bone scintigraphy; CUT, contrastive unpaired translation.

The development environment for the CUT included Python 3.8, PyTorch 1.7.1 and an NVIDIA GeForce RTX 3090 graphics card. For training, we input 195 eBS and 195 dBS images (256 × 1024) into the CUT model as input datasets. Xavier initialization was applied to set the initial weights of the CUT model. Training was performed in unaligned dataset mode with A-to-B translation (eBS to dBS), a batch size of 1, the Adam optimizer (β1 = 0.5 and β2 = 0.999), and an initial learning rate of 0.0002. Because the preprocessing option was set to “none,” no additional study-specific augmentation, such as resizing, cropping, or zooming, was applied; however, the default CUT data pipeline retained random horizontal flipping during training. The adversarial and contrastive loss weights were both set to 1.0 (λGAN = 1.0 and λNCE = 1.0), and identity-based NCE regularization was enabled. Training was conducted for 200 epochs with no decay epochs. Training was conducted separately on the anterior and posterior images of the training dataset, focusing on transforming eBS into dBS. After the training process, 50 anterior and posterior eBS images from the test dataset were input into the trained CUT model, resulting in the generation of 256 × 1024 anterior and posterior aBS images (Figure 1).

### 2.4. Quantitative, Qualitative, and Visual Assessments

In the test datasets, aBS images generated from the CUT were quantitatively, qualitatively, and visually assessed in comparison with eBS and dBS. Quantitative and qualitative assessments were performed separately on the anterior and posterior BS images, whereas visual assessments were conducted on the combined anterior and posterior BS images. The window settings for eBS, dBS, and aBS were adjusted to a window level of 200 and a width of 100. First, we quantitatively compared aBS and eBS results against dBS, using the peak signal-to-noise ratio (PSNR), structural similarity index measure (SSIM), and mean squared error (MSE) to determine superiority. Because eBS and dBS were acquired at different time points and were not pixel-perfectly aligned, PSNR, SSIM, and MSE were used in this study as complementary reference-based metrics under the same test condition rather than as absolute measures of pixel-level fidelity. Specifically, both aBS and eBS were compared against the same dBS reference images, and the quantitative findings were interpreted together with the qualitative and visual assessments [12,13,14,15,16]. Higher PSNR values indicate better image quality. SSIM values increase towards 1 to reflect higher structural similarity [17]. Conversely, as the MSE approaches 0, it indicates better correspondence between the two compared images [12]. The equations for calculating the PSNR, SSIM, and MSE are as follows [18,19].

(1) $$PSNR=20\times {log}_{10}\left(\frac{MAX}{\sqrt{MSE}}\right)$$

(2) $$SSIM=\frac{2\mu_{x}\mu_{y}+C_{1}}{\mu_{x}^{2}+\mu_{y}^{2}+C_{1}}\times \frac{2\sigma_{xy}+C_{2}}{\sigma_{x}^{2}+\sigma_{y}^{2}+C_{2}}$$

(3) $$MSE=\frac{1}{HW}\sum_{i=1}^{H}\sum_{j=1}^{W}{\left(x_{ij}-y_{ij}\right)}^{2}$$

In Equation (1), MAX represents the peak intensity of the images, and MSE stands for mean squared error. In Equation (2), μ_x_ and μ_y_ represent the mean values of the pixel intensities for the comparison and target images, respectively. σ_x_ and σ_y_ represent the standard deviations of the comparison and target images, respectively, while σ_xy_ denotes their covariance. C_1_ and C_2_ are set to 0.01 and 0.03, respectively. In Equation (3), H and W represent the height and width of the image, respectively, while x_ij_ and y_ij_ denote the intensity at pixel (i, j) in the two images. Using Python 3.6.13, along with scikit-image 0.17.2, the PSNR, SSIM, and MSE were computed.

Subsequently, three board-certified nuclear medicine physicians (NMPs) (Y.-J.P., I.-H.K., and S.J.L.) conducted qualitative assessments of the eBS, dBS, and aBS using previously documented four-point and five-point rating scales [20,21]. Visual assessments were conducted on a four-point rating scale for six clinical image criteria: cervical vertebrae, thoracic vertebrae, lumbar vertebrae, thoracic skeleton, pelvic bone, and overall image quality. Assessments were conducted to determine the distinguishability of specific details such as vertebrae and pedicles in the cervical, thoracic, and lumbar vertebrae and the ability to differentiate ribs and their entire lengths in the thoracic skeleton. Furthermore, assessments were made to determine the clarity of the sacroiliac joints, ilium, and pubic bone in the pelvis and to evaluate the overall image quality for excellence. We conducted assessments on image criteria using a four-point rating scale that is outlined as follows: 1 signifies a firm belief that the criterion has not been met; 2 indicates a moderate belief that the criterion has not been met; 3 denotes a moderate belief that the criterion has been met; and 4 implies a firm belief that the criterion has been met [21]. In this study, similar to the five-point rating scale used concurrently, the four-point rating scale was restructured so that higher points corresponded to better evaluations of image quality. In addition to the four-point rating scale, the eBS, dBS, and aBS were evaluated using a five-point rating scale, which is defined as follows: 1, inadequate image quality; 2, nearly adequate image quality; 3, adequate image quality; 4, good image quality; and 5, exceptional image quality for diagnosis [20].

Finally, three board-certified NMPs (Y.-J.P., I.-H.K., and S.J.L.) conducted visual assessments of eBS, dBS, and aBS. Through these assessments, we analyzed the differences between aBS and dBS regarding the presence, location, and number of BM as well as the bone-to-soft tissue contrast among aBS, eBS, and dBS. In this study, dBS was used as the target/reference modality for comparative image assessment rather than as an absolute gold standard. For the three BM-positive patients in the test set, lesion-level interpretability was additionally examined against clinically established BM lesions based on bone biopsy and/or correlative positron emission tomography (PET)/computed tomography (CT) or magnetic resonance imaging (MRI), according to availability in each case. Additionally, we assessed the consistency of patient positioning across aBS, eBS, and dBS and noted any positional differences among the images.

### 2.5. Statistical Analysis

Statistical analyses were performed using the MedCalc software version 22.023 (Ostend, Belgium). Comparisons of the mean values between two continuous variables were conducted using a two-sample *t*-test. Comparisons between two categorical variables were performed using the chi-square test or Fisher’s exact test. Statistical significance was set at a *p*-value < 0.05. For the four- and five-point rating scales, the scores assigned by the three NMPs were averaged for each case, and repeated-measures analysis of variance (ANOVA) was used to compare the mean scores among eBS, dBS, and aBS. When the overall repeated-measures ANOVA was significant, Bonferroni-corrected post hoc pairwise comparisons were performed among eBS, dBS, and aBS. In these pairwise comparisons, a *p*-value < 0.0167 was considered statistically significant. Furthermore, the agreement among three NMPs on the presence of BM, based on visual assessment, was calculated using the kappa coefficient. The kappa coefficient approaches 1 as the agreement between the NMPs becomes almost perfect, indicating a high level of consistency in their assessments.

## 3. Results

### 3.1. Patient Characteristics

Among the 245 patients with BC, 195 (79.6%) and 50 (20.4%) were randomly assigned to the training and test groups, respectively (Table 1). The mean age of the training group (52.6 ± 8.4 years, mean ± standard deviation [SD]) was significantly higher than that of the test group (49.3 ± 7.8 years, mean ± SD) (*p* = 0.0128). All patients in both the training and test groups were confirmed to be women. There was no significant difference in the proportion of patients with BC and BM (*p* = 0.3374) between the training (*n* = 7, 3.6%) and test groups (*n* = 3, 6.0%). Furthermore, there was no significant difference in the waiting time from the intravenous injection of Tc-99m HMDP to the start of imaging with eBS (82.0 ± 13.9 min [training], 81.7 ± 12.1 min [test], *p* = 0.8784) and dBS (202.0 ± 13.5 min [training], 201.4 ± 9.8 min [test], *p* = 0.7842) between the training and test groups. The distribution of gamma camera systems also did not differ significantly between the training and test groups (*p* = 0.8990). A similar result was observed when the systems were grouped by manufacturer (GE Healthcare vs. Siemens Healthcare, *p* = 0.8510).

### 3.2. Quantitative Assessment Results in the Test Datasets

In the test datasets, when comparing aBS and eBS using dBS as reference images, aBS exhibited higher values in PSNR and SSIM than did eBS, while the MSE of aBS was lower than that of eBS (Table 2). Both the anterior and posterior images of aBS demonstrated higher mean PSNR and SSIM values than did those of eBS. Conversely, the mean MSE values of the anterior and posterior aBS images were lower than those of the anterior and posterior eBS images. In an exploratory subgroup analysis across the four gamma camera systems in the test group, no statistically significant differences were observed in PSNR, SSIM, or MSE when aBS and eBS were each compared with dBS as the reference on either anterior or posterior images (Table S1). Given the unpaired nature of the image translation task and the use of dBS as the reference image, these quantitative results were interpreted as comparative findings between aBS and eBS under the same reference condition rather than as absolute measures of pixel-perfect agreement with dBS.

### 3.3. Qualitative Assessment Results in the Test Datasets

In the test dataset, the qualitative assessment results using four-point (Table 3, Figure S2) and five-point rating scales (Table 4, Figure S3) indicated that the image quality of both the anterior and posterior images ranked as follows in descending order: dBS, aBS, and eBS. In the four-point rating scale for both anterior and posterior images, repeated-measures ANOVA test results for dBS, aBS, and eBS demonstrated a significant difference with *p* < 0.001. The Bonferroni-corrected post hoc pairwise comparisons among dBS, aBS, and eBS yielded results with *p* < 0.0001, confirming a *p*-value < 0.0167. The mean five-point rating scale exhibited a similar trend to that observed with the four-point rating scale. In the five-point rating scale applied to both the anterior and posterior images, repeated-measures ANOVA testing for dBS, aBS, and eBS revealed significant differences, with *p* < 0.001. The Bonferroni-corrected post hoc pairwise comparisons among dBS, aBS, and eBS demonstrated significant findings, with *p* < 0.0001, affirming that the *p*-value was indeed < 0.0167. Therefore, in both the four-point and five-point rating scales for the anterior and posterior dBS, aBS, and eBS, a significant difference was observed, with the points gradually decreasing in the order of dBS, aBS, and eBS.

### 3.4. Visual Assessment Results in the Test Datasets

**Figure 2:**
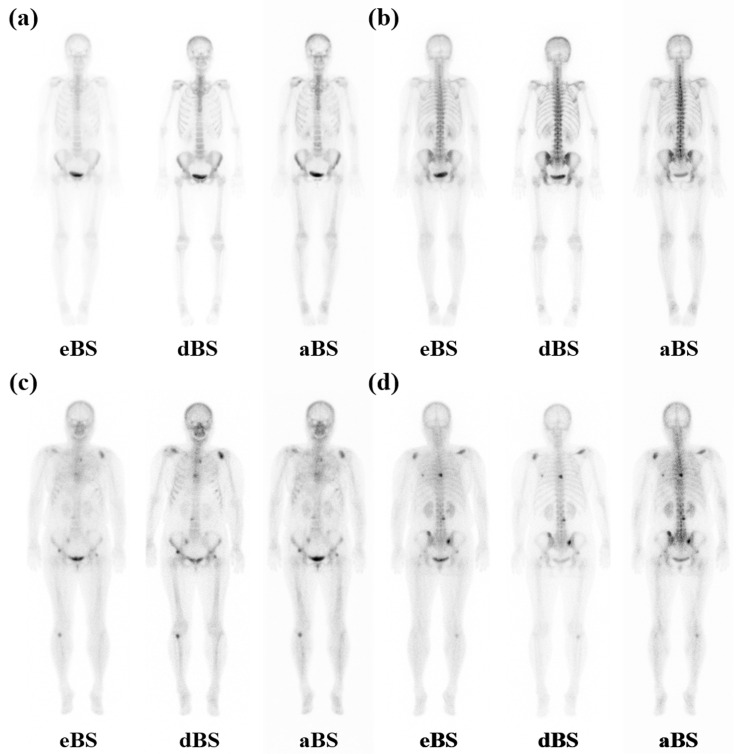
Representative BS images from the test datasets of patients with BC. (**a**,**b**) show a patient without BM, and (**c**,**d**) show a patient with clinically established BM. Anterior and posterior BS images of eBS, dBS, and aBS were presented in cases of patients with and without BM. Abbreviations: BS, bone scintigraphy; BC, breast cancer; BM, bone metastasis; eBS, early-phase bone scintigraphy; dBS, delayed-phase bone scintigraphy; aBS, artificial bone scintigraphy.

In the test group, three NMPs visually assessed the presence of BM in the aBS and dBS. Their evaluations were consistent, with a kappa coefficient of 1. In the test group of 50 patients with BC, visual assessment identified no BM in 47 (94%) patients, whereas BM-related lesions were identified in the remaining three (6%) (Figure 2). Among the three patients with BC and BM, one had a single BM, while the other two had multiple BMs. In these three BM-positive patients, the BM-related uptakes identified on aBS and dBS were concordant in location and number with the clinically established lesions based on bone biopsy and/or correlative PET/CT or MRI, according to availability in each case. Visual assessment of the bone-to-soft tissue uptake contrast among eBS, dBS, and aBS indicated that the contrast was generally better in the order of dBS, aBS, and eBS. While patient positioning in the eBS and aBS images was consistent, different positioning was observed in the dBS images. No obvious artifactual findings that interfered with image interpretation were observed in the generated aBS images from the test datasets. In the 47 BM-negative patients in the test dataset, no BM-like false-positive findings attributable to artificial intelligence (AI) generation were identified on aBS. In particular, no cases were observed in which physiological uptake or motion-related findings on eBS were visually transformed into suspicious BM-like lesions on aBS.

## 4. Discussion

In this single-center prospective study of patients with BC, aBS generated by the unpaired I2I translation method was superior to eBS in quantitative, qualitative, and visual assessments. BM in patients with BC is a major cause of severe morbidity and frequently reduces the quality of life due to immobilization [22,23]. BS is an effective and sensitive method for the initial evaluation and follow-up of BM [24]. However, to achieve the optimal bone-to-background ratio in BS images, scans are typically acquired 3–4 h after intravenous injection of the radiotracer [8]. However, Tc-99m-labeled compounds used in BS present a trade-off between achieving these optimal ratios and rapidly decreasing count rates owing to the 6-h half-life of Tc-99m [6,8]. Moreover, in actual clinical settings, the long waiting time required to obtain dBS images is inconvenient for patients and poses operational challenges for the limited number of gamma cameras in nuclear medicine departments [8]. Therefore, there has been a growing demand to obtain BS with shorter waiting times while preserving clinical information. Recently, advancements in AI methods have led to studies on super-resolution, automatic detection, annotation, diagnosis, classification, and automatic measurement in BS [25,26,27,28,29,30,31,32,33]. Published work directly related to image synthesis in nuclear medicine remains limited but is gradually expanding. In BS, Park et al. used a paired pix2pix framework to translate blood pool images into delayed images in three-phase BS [10]. Related earlier-to-delayed image generation studies have also been reported in fluorine-18 fluorodeoxyglucose PET and Tc-99m methoxyisobutylisonitrile scintigraphy [34,35]. In contrast, the present study focused on whole-body eBS-to-dBS translation in a prospective BC cohort using an unpaired I2I framework. To our knowledge, no peer-reviewed study has directly addressed this specific setting. Within this framework, aBS more closely approximated the imaging characteristics of dBS than did eBS.

In this study, the aBS was significantly superior to the eBS in qualitative assessments using the four-point and five-point rating scales. Khan et al. compared the image quality of eBS (1.5 h) and dBS (3 h) obtained after the injection of Tc-99m MDP [8]. They reported that eBS had fair bone-to-soft tissue contrast, while dBS exhibited good bone-to-soft tissue contrast [8]. These results are consistent with the qualitative assessments performed in our study using four-point and five-point rating scales. A long waiting time to obtain dBS images is necessary to optimize the bone-to-background ratio and enhance the visibility of the target bones [1,7]. As this prolonged waiting time poses challenges, it is necessary to acquire BS images earlier than the recommended 3–4 h post-injection. However, there are theoretical risks associated with reduced image quality and potential missed abnormalities [8]. In this study, we used the CUT model to generate an aBS from an eBS, preserving its structure while learning the appearance or texture of a dBS. The generated aBS was found to be significantly superior to eBS in qualitative assessments, suggesting that aBS contains more dBS imaging characteristics than does eBS. However, the modest quantitative improvements alone do not establish immediate clinical benefit or justify replacement of dBS for definitive diagnosis. Rather, the quantitative findings were deliberately interpreted together with the qualitative ratings and visual assessments, which consistently showed that aBS was closer to dBS than eBS was in the present test set. In this context, the present study is better interpreted as an early proof-of-concept and feasibility study than as a definitive replacement study. Given the still limited published literature on earlier-to-delayed image generation in nuclear medicine, the current findings may provide a basis for subsequent studies evaluating diagnostic performance, workflow impact, and patient-level benefit. However, since aBS was found to be qualitatively inferior to the target dBS on both four-point and five-point rating scales, further research is needed to generate an aBS that is similar to or nearly matches the dBS.

Similar to the qualitative assessments, visual assessments indicated that aBS outperformed eBS and preserved lesion-related information comparable to dBS. Importantly, dBS was not regarded as an absolute gold standard in this study; rather, it served as the target/reference modality for image comparison. In the three BM-positive test cases, BM lesions had been clinically established using bone biopsy and/or correlative PET/CT or MRI, according to availability in each case, and the location and number of BM-related uptakes on aBS were concordant with those clinically established lesions, similar to dBS. Nevertheless, because only three patients with BM were included in the test set, these concordant findings should be interpreted cautiously as preliminary observations rather than definitive evidence of diagnostic accuracy for BM detection. In visual assessments, the bone-to-soft tissue contrast of aBS was better than that of eBS, allowing for better visualization of the bones in aBS than in eBS. Allowing time between injection and imaging permits the bone-seeking radiotracer to clear from soft tissues, leading to a higher bone-to-soft tissue ratio and better bone visualization [36]. Therefore, the visually better bone-to-soft tissue contrast in aBS than in eBS probably reflects a greater clearance of the radiotracer from soft tissues over time in aBS than in eBS. However, because clearance of the radiotracer from soft tissues in aBS was less effective than that in dBS, further research is required to address this issue. In this study, patient positioning in aBS was consistent with that in eBS; however, it differed from that in dBS. In the trained CUT model, eBS was used as the input image, and the application of contrastive loss ensured that the structural integrity and spatial consistency of eBS were preserved in the aBS [11]. Therefore, while aBS had consistent patient positioning with eBS, dBS, which was examined at a different time from eBS, exhibited different patient positioning than both eBS and aBS. Furthermore, owing to the mismatch in structures, such as imaging positions in eBS and dBS, we employed an unpaired I2I translation method, the CUT model. Similar to the qualitative and visual assessments, the quantitative assessments also suggested the superiority of aBS over eBS. However, PSNR, SSIM, and MSE are sensitive to image normalization and spatial translation, and their absolute values should be interpreted cautiously when the reference and comparison images are not perfectly aligned [12,13]. In our study, eBS and dBS were acquired at different time points, and some positional mismatch was unavoidable. Nevertheless, because aBS was generated from eBS and preserved its spatial structure, both the aBS-versus-dBS and eBS-versus-dBS comparisons were performed against the same dBS reference under the same unregistered condition. Therefore, the relative improvement of aBS over eBS remains informative, although these metrics should not be interpreted as definitive evidence of pixel-perfect fidelity. For this reason, we deliberately interpreted the quantitative findings together with the qualitative ratings and visual assessments by three NMPs, rather than relying on PSNR, SSIM, and MSE alone. In addition, prior unpaired I2I studies in medical imaging have also reported PSNR, SSIM, and/or MSE as quantitative evaluation metrics [14,15,16]. Furthermore, the distribution of gamma camera systems between the training and test groups was not significantly different (*p* = 0.8990), and a similar result was observed when the systems were grouped by manufacturer (GE Healthcare vs. Siemens Healthcare, *p* = 0.8510). In addition, exploratory subgroup analysis across the four gamma camera systems in the test group showed no statistically significant differences in PSNR, SSIM, or MSE when aBS and eBS were each compared with dBS as the reference on either anterior or posterior images (Table S1). These findings suggest that the observed superiority of aBS over eBS was not driven by an obvious scanner-specific imbalance within our dataset. At the same time, because the numbers of cases for some camera systems were relatively small, these results should be interpreted cautiously and should not be considered definitive evidence that vendor- or scanner-specific effects are absent. Taken together, these findings support the interpretation that aBS was superior to eBS across qualitative, visual, and complementary quantitative evaluations.

An additional point that warrants consideration is the exclusion of patients with suspected rheumatic or joint disease and suspected trauma from the present cohort. This exclusion was intended to reduce potential confounding from nonmetastatic causes of increased tracer uptake and to allow a more focused proof-of-concept evaluation of whether aBS generated from eBS could reproduce dBS-like imaging characteristics in patients undergoing BM work-up. However, in real-world oncologic practice, degenerative joint disease, inflammatory or infectious skeletal processes, and traumatic bone injury are common causes of increased uptake on BS and may mimic metastatic lesions or produce indeterminate findings, thereby lowering specificity on planar images. In addition, BS can demonstrate increased uptake in arthritic joints, and interpretation may remain challenging even in joints without clearly active clinical diseases. Accordingly, the performance and clinical applicability of the present AI-assisted approach should not be assumed to extend directly to patients with rheumatologic, inflammatory, or traumatic skeletal conditions. Further validation in these clinically relevant subgroups, ideally with correlative imaging and/or standardized reference adjudication, will be necessary to determine whether aBS remains robust when nonmetastatic uptake patterns coexist with or mimic BMs [37,38,39].

Beyond technical performance, the clinical deployment of AI-generated scintigraphic images requires careful ethical, legal, and regulatory consideration. Before routine use, such models should undergo rigorous external and local validation across institutions, scanners, acquisition protocols, and patient populations, ideally with prospective assessment of diagnostic performance, failure modes, and potential bias [40,41]. If AI-generated images are intended to support diagnosis, the software would generally fall under jurisdiction-specific medical device regulation, with pathways such as the Food and Drug Administration premarket review in the United States and Conformité Européenne marking under the European medical device framework, together with requirements for clinical evidence and post-market surveillance [42]. In addition, liability remains an important concern because diagnostic errors associated with AI-generated images may result from model failure, distribution shift, or inappropriate reliance on synthesized images; current legal discussions indicate that responsibility is not transferred entirely to the algorithm and that the interpreting physician may still bear primary clinical accountability [43]. Therefore, AI-generated images should be regarded as assistive tools rather than stand-alone replacements, and review by qualified radiologists or nuclear medicine physicians in conjunction with the original source images remains essential for safe, ethical, and accountable clinical use [42,43].

This study had certain limitations. First, this was a single-center prospective study with a small number of patients with BC, underscoring the need for external validation through multicenter prospective studies involving more diverse patient populations, imaging equipment, and clinical workflows. Second, among the 50 patients with BC in the test group, only three (6%) had BM. Because this was a prospective study conducted over a limited enrollment period, there was an inherent constraint on enrolling a larger number of BM-positive cases. Moreover, because the prospective cohort itself contained only a limited number of BM-positive cases, division of this cohort into training and test datasets inevitably further reduced the number of BM-positive patients available for evaluation in the test set. In addition, a uniform lesion-by-lesion reference standard such as biopsy or correlative PET/CT or MRI was not available for all patients in the test set; therefore, our findings should not be interpreted as definitive validation of diagnostic accuracy against an absolute gold standard. Accordingly, our visual assessment findings regarding the presence, location, and number of BMs should be interpreted as preliminary rather than definitive evidence of diagnostic accuracy for BM detection, and the low prevalence of BM may limit the generalizability of these findings to populations with a higher burden of metastatic disease. Therefore, additional validation of the presence, location, and number of BMs through visual assessment in larger multicenter prospective cohorts, ideally including more patients with BM, is necessary. Third, this study used only one model for unpaired I2I translation, the CUT. Therefore, future studies should explore various unpaired I2I translation methods to identify the optimal model. Fourth, although this study included four different gamma camera systems from two manufacturers and followed the same imaging protocol for all patients, the numbers of cases for some camera systems were relatively small. Although the distribution of gamma camera systems did not differ significantly between the training and test groups and exploratory subgroup analysis in the test group did not show significant differences in PSNR, SSIM, or MSE across the four camera systems, vendor- or scanner-specific effects cannot be completely excluded. Therefore, further validation in larger multivendor and multicenter cohorts is warranted. Fifth, because eBS and dBS were acquired at different time points without image registration, the absolute values of PSNR, SSIM, and MSE may have been influenced by inter-scan spatial mismatch. Although both aBS and eBS were evaluated against the same dBS reference under identical conditions, these quantitative metrics should be interpreted cautiously as complementary rather than definitive measures of pixel-level fidelity. Future studies may benefit from incorporating registration-aware, perceptual, or task-based evaluation strategies [12,13]. Sixth, because dataset allocation was performed using a simple random split without prespecified age-based stratification, the observed age difference between the training and test groups may have contributed to potential confounding related to age-associated skeletal changes, including osteoporosis and degenerative bone changes. Accordingly, the possible influence of these factors on scintigraphic appearance and model evaluation cannot be completely excluded. Seventh, patients with suspected rheumatic or joint disease and suspected trauma were excluded to minimize confounding from nonmetastatic causes of tracer uptake. Although this design facilitated a more focused proof-of-concept evaluation, it may limit generalizability because rheumatologic or other inflammatory skeletal conditions, as well as traumatic bone lesions, can affect BS findings and mimic metastatic disease in real-world practice. Therefore, further validation in these clinically relevant subgroups is warranted.

## 5. Conclusions

In this single-center prospective study of patients with BC, aBS generated through unpaired I2I translation was superior to eBS in quantitative, qualitative, and visual aspects, supporting the feasibility of further investigating this approach. The aBS images more closely approximated the imaging characteristics of dBS than eBS and showed preservation of lesion-related information comparable to dBS. Although these findings do not support replacement of dBS for definitive diagnosis, they suggest that aBS may have value as an assistive delayed-phase-like image generation approach and may provide a basis for future studies evaluating diagnostic performance, workflow impact, and patient-level benefit. At the same time, aBS remained inferior to the target dBS in qualitative and visual assessments, underscoring the need for further research to improve aBS.

## Figures and Tables

**Table 1:** Patient characteristics in the training and test datasets of patients with BC.

		Training Datasets (*n* = 195)		Test Datasets (*n* = 50)		
		Mean ± SD	Number (%)	Mean ± SD	Number (%)	*p*-Value
Age (year)		52.6 ± 8.4		49.3 ± 7.8		0.0128 ^a,b^
Sex (female)			195 (100)		50 (100)	1.0000 ^c^
BM			7 (3.6)		3 (6.0)	0.3374 ^c^
Waiting time of eBS (minutes)		82.0 ± 13.9		81.7 ± 12.1		0.8784 ^b^
Waiting time of dBS (minutes)		202.0 ± 13.5		201.4 ± 9.8		0.7842 ^b^
Gamma cameras						
	Infinia Hawkeye 4		77 (39.5)		21 (42.0)	0.8990 ^d^
	Symbia E		65 (33.3)		18 (36.0)	
	NM 830		13 (6.7)		3 (6.0)	
	NM/CT 670		40 (20.5)		8 (16.0)	

Abbreviations: BC, breast cancer; SD, standard deviation; BM, bone metastasis; eBS, early-phase bone scintigraphy; dBS, delayed-phase bone scintigraphy. ^a^ *p* < 0.05. ^b^ Two-sample *t*-test. ^c^ Fisher’s exact test. ^d^ Chi-squared test.

**Table 2:** Quantitative results for aBS and eBS against dBS as the reference in the test datasets.

		PSNR ↑	SSIM ↑	MSE ↓
		(dB)		(squared units)
		Mean	Mean	Mean
Anterior	aBS	**27.0825**	**0.8485**	**149.8190**
	eBS	26.6365	0.8362	175.0457
Posterior	aBS	**28.4433**	**0.8449**	**112.4829**
	eBS	27.7058	0.8429	146.4943

Abbreviations: aBS, artificial bone scintigraphy; eBS, early-phase bone scintigraphy; dBS, delayed-phase bone scintigraphy; PSNR, peak signal-to-noise ratio; SSIM, structural similarity index measure; MSE, mean squared error.

**Table 3:** Results of the four-point rating scale for eBS, aBS, and dBS in the test datasets.

	eBS	aBS	dBS	Overall	eBS vs. aBS	eBS vs. dBS	aBS vs. dBS
	Mean	Mean	Mean	*p*-Value ^a^	Adj. *p*-Value ^b^	Adj. *p*-Value ^b^	Adj. *p*-Value ^b^
Four-point rating scale							
Anterior	1.9267	2.6800	3.8933	<0.001 ^c^	<0.0001 ^d^	<0.0001 ^d^	<0.0001 ^d^
Posterior	2.5667	3.4067	3.9933	<0.001 ^c^	<0.0001 ^d^	<0.0001 ^d^	<0.0001 ^d^

Abbreviations: eBS, early-phase bone scintigraphy; aBS, artificial bone scintigraphy; dBS, delayed-phase bone scintigraphy; ANOVA, analysis of variance. ^a^ Repeated-measures ANOVA. ^b^ Bonferroni-corrected post hoc pairwise comparisons. ^c^ *p* < 0.05. ^d^ Adjusted *p* < 0.0167.

**Table 4:** Results of the five-point rating scale for eBS, aBS, and dBS in the test datasets.

	eBS	aBS	dBS	Overall	eBS vs. aBS	eBS vs. dBS	aBS vs. dBS
	Mean	Mean	Mean	*p*-Value ^a^	Adj. *p*-Value ^b^	Adj. *p*-Value ^b^	Adj. *p*-Value ^b^
Five-point rating scale							
Anterior	2.3467	3.2333	4.8400	<0.001 ^c^	<0.0001 ^d^	<0.0001 ^d^	<0.0001 ^d^
Posterior	3.1067	4.0600	4.9667	<0.001 ^c^	<0.0001 ^d^	<0.0001 ^d^	<0.0001 ^d^

Abbreviations: eBS, early-phase bone scintigraphy; aBS, artificial bone scintigraphy; dBS, delayed-phase bone scintigraphy; ANOVA, analysis of variance. ^a^ Repeated-measures ANOVA. ^b^ Bonferroni-corrected post hoc pairwise comparisons. ^c^ *p* < 0.05. ^d^ Adjusted *p* < 0.0167.

## Data Availability

The data generated or analyzed during the present study are not publicly available due to ethical and legal restrictions related to patient privacy but may be available from the corresponding author upon reasonable request, subject to institutional approval.

## Supplementary Materials

The following supporting information can be downloaded at: https://www.mdpi.com/article/10.3390/tomography12040050/s1, Figure S1: Training loss curves of the CUT model used to generate aBS; Figure S2: Comparison of the mean points on the four-point rating scale in the test datasets; Figure S3: Comparison of the mean points on the five-point rating scale in the test datasets; Table S1: Comparisons of quantitative metrics derived from aBS and eBS using dBS as the reference in the test group, across four different gamma cameras.

## Abbreviations

The following abbreviations are used in this manuscript:

aBS	Artificial bone scintigraphy
AI	Artificial intelligence
ANOVA	Analysis of variance
BC	Breast cancer
BM	Bone metastasis
BS	Bone scintigraphy
CRPS	Complex regional pain syndrome
CT	Computed tomography
CUT	Contrastive unpaired translation
dBS	Delayed-phase bone scintigraphy
eBS	Early-phase bone scintigraphy
GAN	Generative adversarial network
HMDP	Hydroxymethylene diphosphonate
I2I	Image-to-image
MDP	Methylene diphosphonate
MRI	Magnetic resonance imaging
MSE	Mean squared error
NMP	Nuclear medicine physician
PET	Positron emission tomography
PSNR	Peak signal-to-noise ratio
SD	Standard deviation
SSIM	Structural similarity index measure
Tc-99m	Technetium-99m
