# Supplementary material for: Clinical Potential of Artificial Bone Scintigraphy from Early-Phase Bone Scintigraphy Using Unpaired Image-to-Image Translation in Patients with Breast Cancer: A Single-Center Prospective Study
Source: Tomography. 2026 Apr 2;12(4):50. doi: 10.3390/tomography12040050 (PMC13119574; doi:10.3390/tomography12040050)
Supplement: Supplementary file 1 [file tomography-12-00050-s001.zip › tomography-4174746-supplementary.pdf]

<Supplementary Materials>

(a)

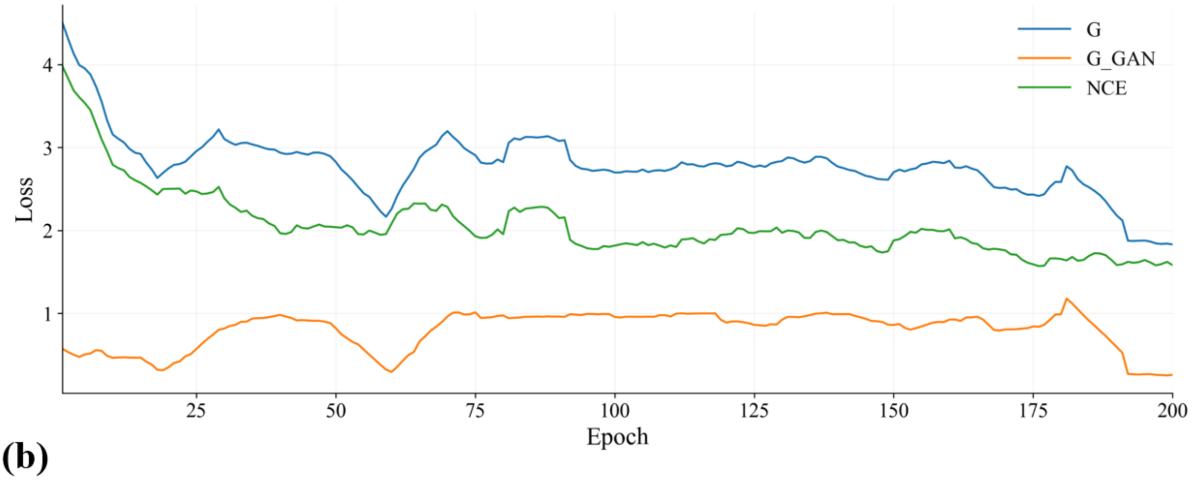

(b)

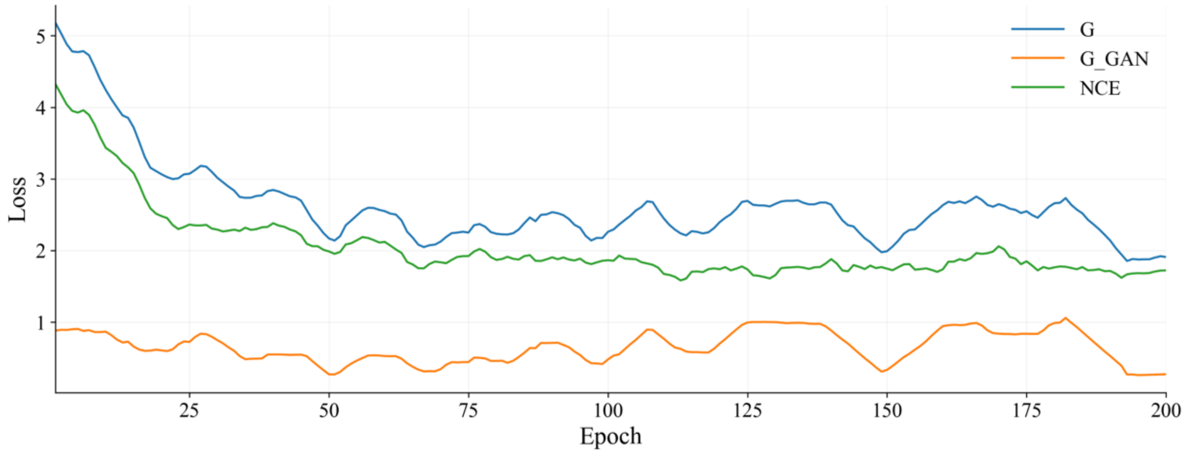

Figure S1. Training loss curves of the CUT model used to generate aBS. Panel (a) shows the anterior model, and panel (b) shows the posterior model. Epoch-wise mean values of the overall generator loss (G), adversarial generator loss (G_GAN), and contrastive loss (NCE) monitored during training are shown.

Abbreviations: CUT, contrastive unpaired translation; aBS, artificial bone scintigraphy.

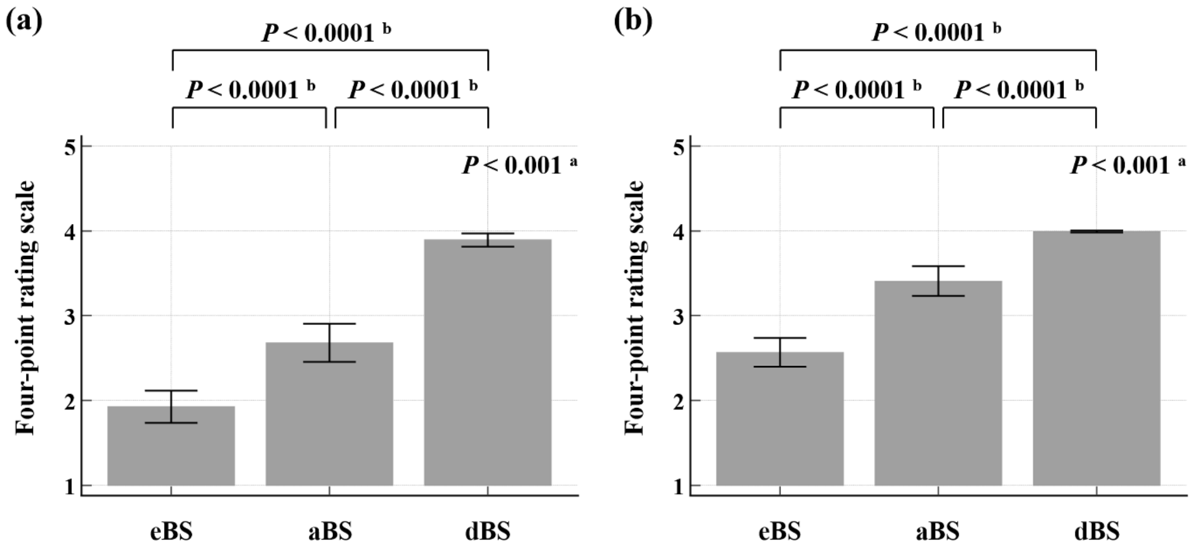

Figure S2. Comparison of the mean points on the four-point rating scale in the test datasets. The mean points on a four-point rating scale were evaluated by three NMPs for (a) anterior and (b) posterior eBS, aBS, and dBS within the test datasets. Bar graphs depicted the mean points on the four-point rating scale for eBS, aBS, and dBS, with error bars showing the 95% confidence intervals.

Abbreviations: NMP, nuclear medicine physician; eBS, early-phase bone scintigraphy; aBS, artificial bone scintigraphy; dBS, delayed-phase bone scintigraphy; ANOVA, analysis of variance.

[a] $P < 0.05$ in the repeated-measures ANOVA

[b] $P < 0.0167$ after Bonferroni correction for post hoc pairwise comparisons

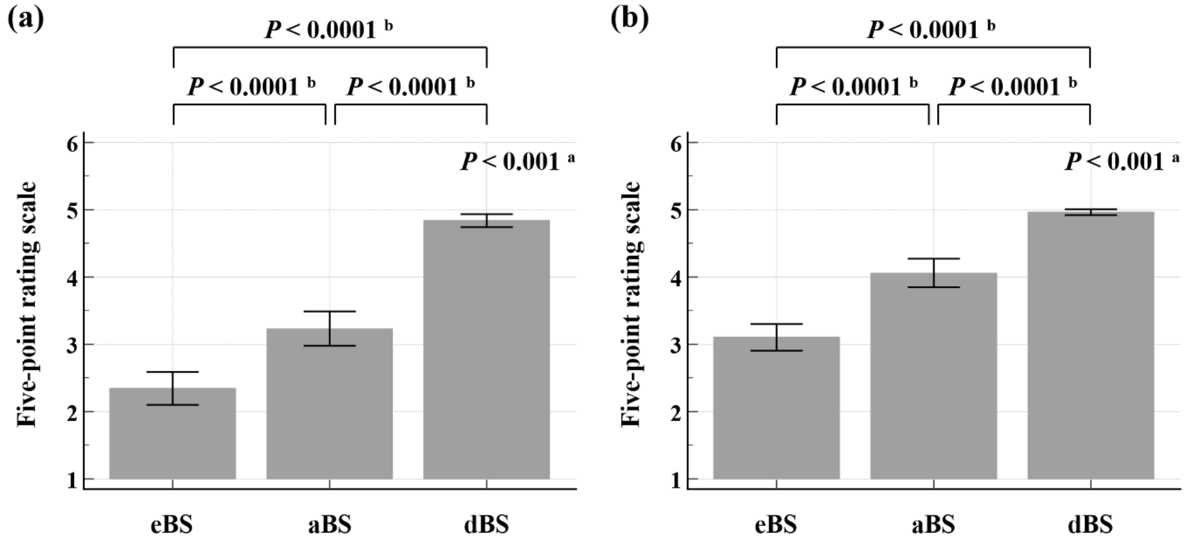

Figure S3. Comparison of the mean points on the five-point rating scale in the test datasets. The mean points on a five-point rating scale were evaluated by three NMPs for (a) anterior and (b) posterior eBS, aBS, and dBS within the test datasets. Bar graphs depicted the mean points on the five-point rating scale for eBS, aBS, and dBS, with error bars showing the 95% confidence intervals.

Abbreviations: NMP, nuclear medicine physician; eBS, early-phase bone scintigraphy; aBS, artificial bone scintigraphy; dBS, delayed-phase bone scintigraphy; ANOVA, analysis of variance.

[a] $P < 0.05$ in the repeated-measures ANOVA

[b] $P < 0.0167$ after Bonferroni correction for post hoc pairwise comparisons

Table S1. Comparisons of quantitative metrics derived from aBS and eBS using dBS as the reference in the test group, across four different gamma cameras

			P value [a]
Anterior	aBS	PSNR	0.704
		SSIM	0.819
		MSE	0.662
	eBS	PSNR	0.605
		SSIM	0.690
		MSE	0.448
Posterior	aBS	PSNR	0.733
		SSIM	0.855
		MSE	0.712
	eBS	PSNR	0.268
		SSIM	0.812
		MSE	0.279

Abbreviations: aBS, artificial bone scintigraphy; eBS, early-phase bone scintigraphy; dBS, delayed-phase bone scintigraphy; PSNR, peak signal-to-noise ratio; SSIM, structural similarity index measure; MSE, mean squared error; ANOVA, analysis of variance.

[a] One-way ANOVA
